# Utilization of Waste Leather Powders for Highly Effective Removal of Dyes from Water

**DOI:** 10.3390/polym11111786

**Published:** 2019-11-01

**Authors:** Liangjun Xia, Chen Li, Sijie Zhou, Zhuan Fu, Yun Wang, Pei Lyu, Jiajing Zhang, Xin Liu, Chunhua Zhang, Weilin Xu

**Affiliations:** 1 State Key Laboratory of New Textile Materials and Advanced Processing Technologies, Wuhan Textile University, Wuhan 430200, China; liangjun_xia@hotmail.com (L.X.); 15927512243@163.com (C.L.); sijie_zhou9602@163.com (S.Z.); zhuan_fu1996@163.com (Z.F.); 15527072729@163.com (Y.W.); zjj18672519077@163.com (J.Z.); xinliu_wtu@163.com (X.L.); 2 Institute for Frontier Materials, Deakin University, Geelong, Victoria 3216, Australia; lyup@deakin.edu.au; 3 College of Material Science and Engineering, Wuhan Textile University, Wuhan 430073, China

**Keywords:** natural polymer, leather waste, powder, dyes, adsorption, wastewater

## Abstract

As a natural polymer, leather and its associated industries are known to be the leading economic sector in many countries. However, the huge amounts of leather waste generated from the leather industry causes severe environmental pollution. Herein, cow leather (CL) powders were prepared using a homemade machine and used as a low-cost adsorbent for the effective removal of reactive dyes from wastewater. The as-prepared CL powders exhibited dot-like, rod-like, and fiber-like morphologies. A Fourier transform infrared analysis and an x-ray diffraction analysis demonstrated that the CL powders retained the main structure of the protein contained in it. In addition, an improvement in thermal stability was also observed for the CL powders. Dye adsorption experiments indicate that the CL powders showed the highly effective removal of C.I. Reactive Red 120 (RR120), C.I. Reactive Yellow 127 (RY127), and C.I. Reactive Blue 222 (RB222) with the adsorption capacity of 167.0, 178.9, and 129.6 mg·g^−1^, respectively. The Langmuir, pseudo-second order, and intraparticle diffusion models could well depict the adsorption equilibrium and kinetics of CL powders toward the investigated reactive dyes. The as-prepared CL powders can be used as a potential adsorbent in the treatment of dye contaminated wastewater. Future studies will mainly focus on the application of the adsorbed CL powders for the pigment printing of textile materials.

## 1. Introduction

Water security is certain to be a major topic facing humanity. It has been reported that the freshwater ecosystem only occupies nearly 1% of the Earth’s surface [1]. Hence, as the global population increases, enormous stress on the requirement of freshwater for both people and nature will be generated yearly [2]. Additionally, rising industrialization, especially that of the textile industry, consumes large amounts of water and discharges wastewater with deep colors [3,4]. Although we developed an environmentally friendly dyeing method to conserve water consumption and mitigate water pollution in our previous study [5], the elimination of dyes in conventional textile effluents is still of great importance to the whole textile industry.

Recently, various technologies such as adsorption [6,7,8,9], photochemical degradation [10,11], membrane filtration [12,13], and biological treatment [14,15] have become available for color removal from dye contaminated water. Based on sustainable development, adsorption using waste materials has been considered to be the most promising technology among these approaches [16].

Leather products in the form of protein are widely used in daily human life [17]. However, it is reported that nearly 30% of the leftover leather materials processed in tanneries is abandoned [18]. In addition, statistical data on the leather industry shows that nearly 0.15 million tons of leather solid-waste is produced annually in India, and 1.4 million tons in China [17]. The arbitrary disposal of leather shavings and products inevitably results in great damage to the environment, but also represents a considerable waste of existing resources. Therefore, the reuse of leather waste, and in particular secondary waste, has become an important research problem.

Significant research and attention in a number of publications has focused on the isolation and production of collagens from leather waste, and on more detailed reviews of the pretreatment method such as chemical treatment (oxidation of hydrolyzes) and enzymatic digestion [17,19]. An increasing number of studies consider the incorporation of leather as a filler in polymers including rubbers (natural or synthetic) [20,21,22], poly (vinyl butyral) [23], and polyamide [24]. Moreover, a series of methods for processing leather waste including waste dumps [17], combustion [25], pyrolysis [26], carbonization [27] fertilizer [28], and pigments [29] have been extensively employed to treat leather waste. However, most of these methods are likely to generate secondary pollutants, and can only be applied in limited application areas, which may put considerable pressure on highly fragile ecological environments.

Powder, a popular and low-cost technology, has been proven to be an effective and economical absorbent for removing dyes from wastewater [8,16,30]. In our previous studies, silk fibroin powder [16], down powder [31,32], and needle down powder [33] were examined in terms of their capacity to remove dyes from dyeing effluents. However, to the best of our knowledge, there have only been a few reported studies on the preparation of leather powder and its applications in dye removal, which is mainly due to its unique structure containing strong hydrogen and ionic bonding, thus making it difficult to grind into fine or superfine powder without high-pressure homogenization or chemical pretreatment.

In this paper, a homemade instrument [34,35], which can be used to grind both primary and secondary leather waste into powders at room temperature and atmospheric pressure without chemical pretreatment, was employed to produce the leather powder. In addition, the morphologies, microstructures, and thermo-gravimetric analysis were considered to understand the nature of this leather. Finally, three kinds of reactive dyes including C.I. Reactive Red 120 (RR120), C.I. Reactive Yellow 167 (RY167), and C.I. Reactive Blue 222 (RB222) were used to examine the removal capacity of leather powder. The effects of pH value, contact time, and the adsorption isotherm and kinetic models were systematically studied. This is the first-time that leather powder has been investigated in the application of dye removal from wastewater.

## 2. Materials and Methods

### 2.1. Materials

**Figure 1:**
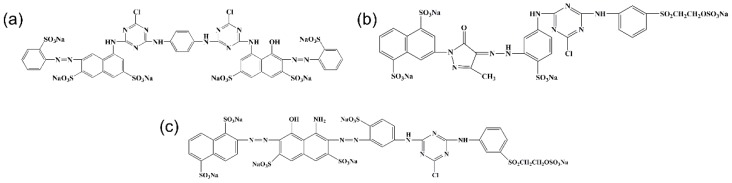
Chemical structures of (**a**) RR120, (**b**) RY167, and (**c**) RB222, respectively.

Waste cow leather (CL) was supplied by a local tannery in Zhejiang Province, China. Acetic acid (HAC), sodium hydrogen phosphate (Na_2_HPO_4_·12H_2_O), citric acid monohydrate (C_4_H_2_O_7_·H_2_O), Sodium hypochlorite (NaClO), and absolute ethanol were purchased from Sinopharm Chemical Reagent Co. Ltd., Shanghai, China. The RR120, RY167, and RB222 dyes were kindly supplied by Luthai Textile Co. Ltd., Shandong, China, and the chemical structures of the dyes are shown in Figure 1. Deionized water was employed throughout the experiments. All chemicals were of analytical grade and used as received.

### 2.2. Preparation of CL Powder

**Figure 2:**
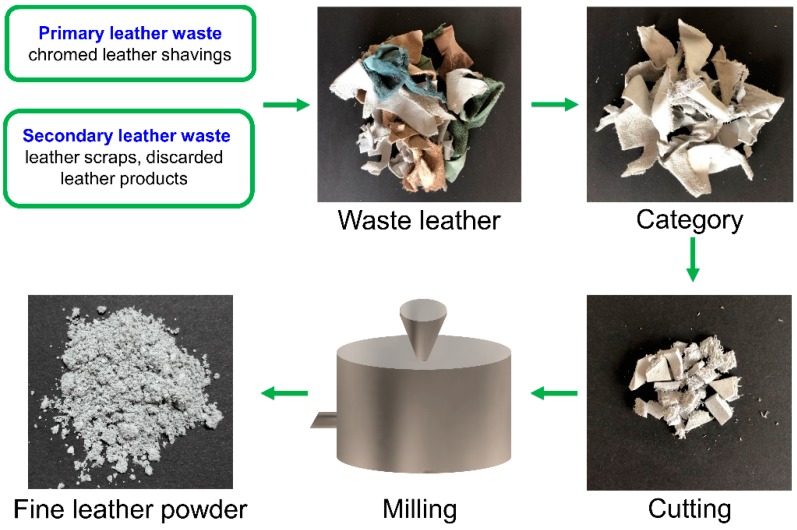
Preparation procedure for the CL powder.

The preparation procedures of the CL powders are illustrated in Figure 2. Typical procedures were performed by categorizing the waste leathers into different colors. In this experiment, the waste leathers with white or light blue colors were selected for the removal of dyes from water. Then, the categorized waste leathers were disinfected using a 0.5% NaClO solution at room temperature. After the residual moisture of the waste leather was removed, the leather was cut into short pieces (ca. 10 mm) using a rotary blade, and then ground between two milling pans using a special method that employed a homemade instrument, which was previously reported in our studies for the preparation of superfine wool and down powder [34,36]. The mills had special properties such as very low heat generation and high anti-abrasion properties. The leather pieces could be crushed into fine leather powder under the mechanical action, which included pressure, drawing, torsion, and sheer action.

### 2.3. Adsorption Study

Typically, reactive dyes play an essential role in the textile dyeing industry [37], which occupy more than 30% of the total dye market share, and this number is increasing dramatically [38]. Therefore, in the present study, three kinds of dyes (RR120, RY167, and RB222) that are usually employed in the cotton dyeing industry were selected as representative dyes to prepare the dye-contaminated wastewater. The pH of the dye solutions was adjusted using HAC, Na_2_HPO_4_·12H_2_O, and C_4_H_2_O_7_·H_2_O. A series of batch experiments were conducted by placing 0.3 g of CL powders evenly inside the conical flasks filled with 50 mL of different dye solutions at room temperature (30 °C). The parameters of pH (3–8), contact time (0–100 min), and dye concentration (50–1200 mg·L^−1^) were investigated to examine the adsorption properties of CL powder toward the three dyes.

The dye removal efficiency (% RE) and the adsorption capacity at equilibrium (*Q*_e_, mg·g^−1^) was calculated using Equations (1) and (2) [39,40], respectively.

(1) $$RE=\frac{\left(C_{0}-C_{e}\right)}{C_{0}}\times 100\%$$

(2) $$Q_{e}=\frac{(C_{0}-C_{e})V}{m}$$

where *C*_0_ and *C_e_* are the initial and equilibrium concentrations of the dye solutions (mg·L^−1^), respectively; *V* is the volume of dye solution (L); and *m* is the mass of the CL powder.

### 2.4. Characterization

Photographs of the CL and CL powder were measured using a digital camera (Canon EOS 80D). The maximum Feret diameter (MFD) of CL powder was observed using optical microscopy (CKX41, Olympus Co., Tokyo, Japan). The CL powder was ultrasonically dispersed in an ethanol–water mixture (90:10 wt.%). Then, the CL powder solution was placed onto a glass slide for observation. The typical morphology of the CL powders was revealed using Image Pro Plus (edition 6.0). Morphologies of the CL and CL powder were examined using scanning electron microscopy (SEM) (JSM-6510LV, JEOL Ltd., Tokyo, Japan). Samples were sputter-coated with a thin gold layer before testing. Dynamic light scattering (DLS) measurements were used to examine the mean zeta potential (ZP) analyses of the dyes and CL powders using a Zetasizer ZEN3600 instrument (Malvern Instruments Ltd., Malvern, UK) at room temperature. Fourier transform infrared spectroscopy (FTIR) of the CL and CL powder were characterized on a Bruker LUMOS FTIR microscope (Bruker corporation, Karlsruhe, Germany). FTIR was employed to record the spectra of the films from 4000 to 1000 cm^−1^ with 256 scans at the resolution of 4 cm^−1^. All measurements were repeated three times from three different spots on the same sample. X-ray diffraction (XRD) was performed on an X’pert Pro, PANalytical B.V., Holland using Cu kα radiation where the irradiation conditions were 40 kV and 40 mA. Samples were scanned from 5 to 70° (2θ) with a step length of 0.02°. Thermo-gravimetric analysis (TGA) and derivative thermogravimetric (DTG) curves were obtianed by using NETZSCH TG 209 F1 (Netzsch Ltd., Hanau, Germany) in flowing nitrogen gas (20 mL·min^−1^). The samples were heated from 25 °C up to 800 °C with a heating rate of 10 °C·min^−1^.

## 3. Results and Discussion

### 3.1. Characterization of CL and CL Powder

**Figure 3:**
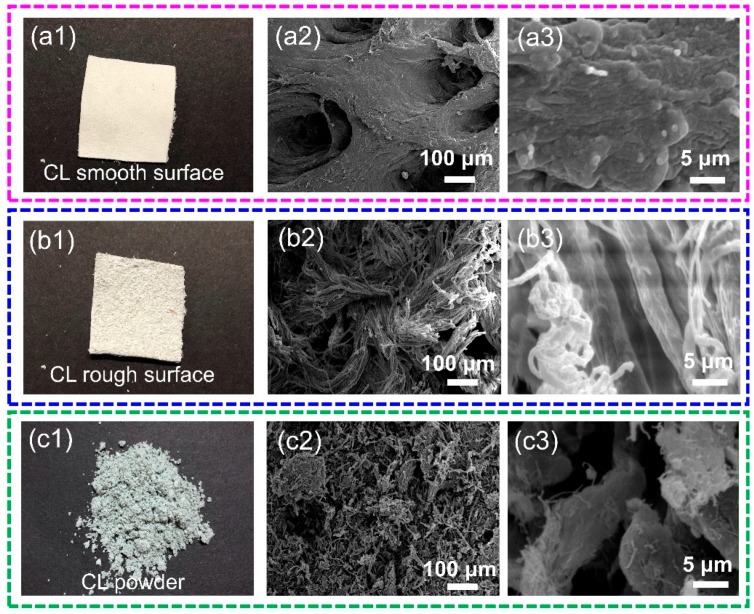
(**a1**–**a3**) Photographs and SEM images for the smooth surface of CL; (**b1**–**b3**) photographs and SEM images for the rough surface of CL; (**c1**–**c3**) photographs and SEM images of CL powders.

The morphologies of CL and the CL powder were examined using SEM. As shown in Figure 3, the photographs and micrographs exhibited significant changes in the morphologies of the CL and CL powder. The images in Figure 3a revealed that the smooth surface of the CL was compact, indicating that it is difficult to triturate CL into powder. In addition, various irregular shaped fibrils were present on the opposite side of the smooth surface of the CL (Figure 3b). Figure 3c shows that the CL was crushed into very small particles, especially in diameter, and most of these exhibited fiber-like and rod-like morphologies with a diameter of 5–10 μm (obtained from ImageJ Pro Plus).

The measurement of particle sizes varies in complexity depending on the shape of the particle. Generally, the particle size of a spherical object can be unambiguously and quantitatively defined by its diameter, due to the same size in different orientations (0–180 degree). For irregular particles, Feret’s diameter is a frequently used descriptor for the evaluation of particle size distribution, according to previous literature [41,42,43,44,45]. The maximum Feret’s diameter (MFD) is defined is the longest distance between two points on the perimeter between which a line can be drawn within the perimeter.

**Figure 4:**
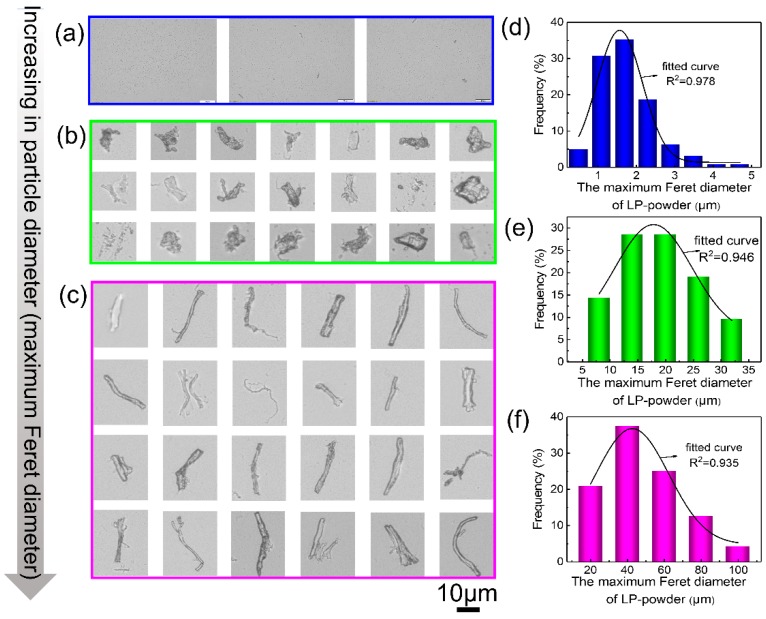
(**a**–**c**) Morphologies of the dot-like (**a**), rod-like (**b**), and fiber-like (**c**) particles; (**d**–**f**) histograms of the maximum Feret diameter of the dot-like (**d**), rod-like (**e**), and fiber-like (**f**) particles.

Optical microscope images of CL powders revealed a different morphology, shape, and size of the particles, which can be classified into three types: dot-like particles, rod-like particles, and fiber-like particles, respectively (as shown in Figure 4a–c). This could be ascribed to the structural inconsistency of CL (CL smooth surface and CL rough surface), which might enable uniform force during milling by using a homemade instrument. To accurately describe the morphological features of the CL powders, numerous particles for each shape were analyzed from the optical microscope images and the value of the averaged MDF were calculated. As depicted in Figure 4, dot-like particles had an average MFD of 1.6 μm, rod-like particles had an average MFD of 17.8 μm, and fiber-like particle had an average MFD of 40.1 μm, respectively. The morphologies of the CL powder were consistent with the SEM images.

**Figure 5:**
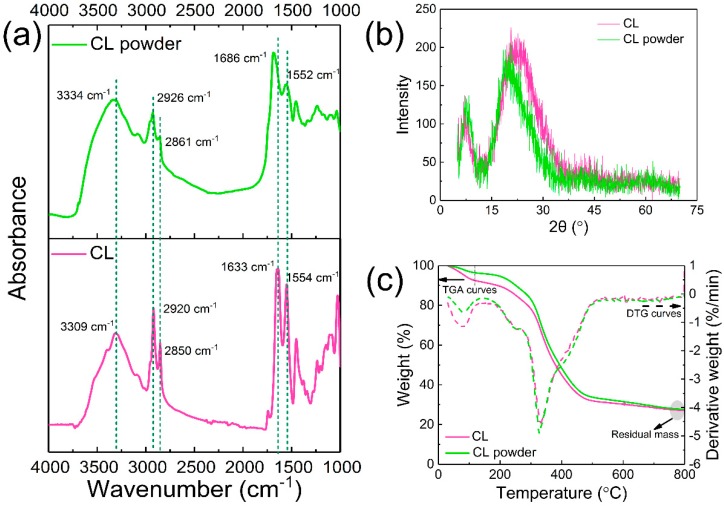
(**a**) FTIR spectra of CL and CL powder; (**b**) XRD curves of CL and CL powder; (**c**) TGA (left) and DTG (right) curves of CL and CL powder.

Figure 5a shows the FTIR spectra of the CL and CL powder. The CL exhibited characteristic peaks at 3306 cm^−1^ (the combined effect of N–H and O–H stretching vibrations), 2920 cm^−1^ (C-H asymmetric stretching vibrations), 2850 cm^−1^ (C–H symmetric stretching vibrations), 1633 cm^−1^ (amide I), and 1554 cm^−1^ (amide II). These bands are similar to the FTIR spectra of protein fibers such as silk, wool, and down fibers due to the similarities in the chemical structures of keratins [34,35]. In comparison, the CL powder exhibited characteristic peaks at 3309 cm^−1^, 2926 cm^−1^, 2861 cm^−1^, 1686 cm cm^−1^, and 1552 cm cm^−1^, indicating the main structure of keratins in the CL powder. Furthermore, no new bands could be seen in the spectra of the CL powder, suggesting that the milling process does not produce new bands. The sharper peak at 3309 cm^−1^ shifted to 3334 cm^−1^ (broad peak) in Figure 5a. This shift change confirms the transformation of strong hydrogen bonds to weak hydrogen bonds due to the milling process [35,46]. Meanwhile, the band of amide I (1633 cm^−1^) shifted to a higher wavenumber (1686 cm^−1^) after milling. These two peaks can be ascribed to the β-sheet [47]. The FTIR spectra of CL exhibited more significant β-sheet conformation peaks in amide I and amide II than the CL powder, which indicates some changes in the C=O groups.

To investigate the effect of the developed method on the intensity of CL, x-ray diffraction was performed. As shown in Figure 5b, all x-ray diffractograms exhibited a minor peak at 7.9° (2*θ* angle), a broad peak at 22° (2*θ* angle), and a minor peak at 29.7°. The first intense peak at 7.9° for the CL and CL powder was consistent with the leather shavings in the studies of Li et al. [48]. As depicted in Figure 5b, the intensity of the peak at 7.9° for the CL remained the same after grinding. Furthermore, the peak intensities of the CL powder (a broad peak around 22°) were lower than for the CL, indicating a slight decrease in the crystallinity of the CL. This minor reduction could be ascribed to the crystal being destroyed in the milling process. Moreover, both the CL and CL powder maintained the triple helical structure located at around 27.90°. Thus, the CL powder could maintain its original structure after milling.

The TGA and DTG curves for the CL and CL powder are presented in Figure 5c. The TGA curve for the CL powder was similar to that of the CL, which exhibited a two-stage degradation. The first stage of degradation (stage-I), between 35 and 120 °C, can be attributed to water evaporation and small molecular substances. The second stage of degradation (stage-II), between 120 and 800 °C, is associated with the decomposition of peptide bonds [34,49]. As depicted in Figure 5c, the weight loss of the absorbed water, bound water, and small molecular substances in CL was about 8.5% (stage-I). For the CL powder, the water content was reduced to 4.1% when compared with the CL. This decrease can be ascribed to the volatilization of small molecular substances and the destruction of the polar groups during milling. Meanwhile, the decomposition temperature plotted in the DTG curves was about 330 °C for both the CL and CL powder, and the residual of the CL powder (27.6%) increased when compared with the CL (27.0%). According to our previous research, another natural protein powder (superfine down powder) fabricated using this method exhibited the same trend for the enhancement of the residual (TGA in a N_2_ atmosphere) [50,51]. Therefore, the enhancement in thermal stability could be attributed to the higher relative presence of retained carbon elements in the CL powder when compared to the noncarbon elements such as NH_3_ lost during the milling process.

### 3.2. Adsorption Isotherms

**Figure 6:**
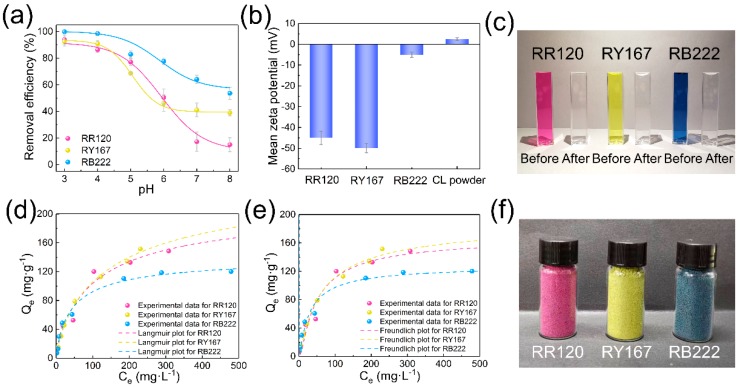
(**a**) Effect of pH on the adsorption of RR120, RY167, and RB222 onto the CL powders (m = 0.3 g, V = 50 mL, C_0_ = 50 mg·L^−1^, T = 30 °C, T = 100 min); (**b**) mean zeta potential of RR120, RY167, RB222, and CL powders, respectively; (**c**) photograph of the dye solution before and after the adsorption by the CL powders at the pH of 3.0 and initial concentration of 50 mg·L^−1^; (**d**) Langmuir and (**e**) Freundlich adsorption isotherms for the adsorption of RR120, RY167, and RB222 by the CL powders (m = 0.3 g, V = 50 mL, pH = 3.0, T = 30 °C); (**f**) photograph of the CL powders after the adsorption of RR120, RY167, and RB22, respectively.

Solution pH significantly affects the adsorption performance of an adsorbent, especially the surface charge of the adsorbent and the dissociation degree of the dye molecule [8]. The curves in Figure 6a show the influence of solution pH on the adsorption of RR120, RY167, and RB222 onto CL powder within the pH range of 3.0–8.0 at an initial concentration of 50 mg·L^−1^. The maximum adsorption efficiency occurred at a pH of 3.0 for all of the investigated dyes, which were 94.0%, 92.5%, and 99.9% for RR120, RY167, and RB222 at an initial dye concentration of 50 mg·L^−1^, respectively. This can be ascribed to the dissociation of these anionic dyes in the aqueous solution [8]. Furthermore, as shown in Figure 6b the electrostatic attraction between the positively charged CL powder and the negatively charged dye molecule were significantly enhanced under acidic conditions [39], resulting in the high removal efficiency.

The adsorption isotherm is generally applied to elucidate the relationship between the adsorbent and dyes under equilibrium conditions. Moreover, the maximum adsorption capacities of the adsorbent toward dyes can be calculated according to the adsorption isotherm [52]. In this study, two well-known Langmuir and Freundlich adsorption isotherms were employed to obtain deeper insight into the removal of reactive dyes by the CL powders. The Langmuir isotherm describes the single layer adsorption on a homogeneous surface of the adsorbent [52], which can be expressed as Equation (3) [53,54],

(3) $$\frac{C_{e}}{Qe}=\frac{1}{K_{l}Q_{m}}+\frac{C_{e}}{Q_{m}}$$

where *C_e_* is the equilibrium concentration of the reactive dyes (mg·L^−1^); *Q_e_* is the equilibrium adsorption capacity of the reactive dyes adsorbed onto the CL powders (mg·L^−1^); *Q_m_* (mg·L^−1^) represents the maximum adsorption capacity; and *K_l_* (L·mg^−1^) is a constant of the Langmuir isotherm.

Furthermore, the basic characteristic of the Langmuir isotherm model can be expressed by the dimensionless differentiation factor (*R*_l_), which is defined as Equation (4) [53],

(4) $$R_{l}=\frac{1}{1+K_{l}C_{0}}$$

Herein, the adsorption is typically considered irreversible (*R*_l_ = 0), favorable (0 < *R*_l_ < 1), linear (*R*_l_ = 1), or unfavorable (*R*_l_ > 1), respectively.

Additionally, the Freundlich isotherm explains the multilayer adsorption on a heterogeneous surface relating to varied non-ideal conditions [52], which can be expressed as Equation (5) [53,54],

(5) $$\ln Q_{e}=lnK_{f}+\frac{1}{n}lnC_{e}$$

where *K_f_* and n are empirical constants of the Freundlich adsorption model

Figure 6d,e show the Langmuir and Freundlich adsorption isotherms of the reactive dyes onto the CL powders, respectively. The adsorption capacity generally affects the practical application of an adsorbent [55], which can be estimated according to the Langmuir and Freundlich isotherm models [53,56]. Therefore, the values of *Q_m_* and *K_l_* from the Langmuir isotherm model were calculated according to the slope and intercept for the linear plot of *C_e_/Q_e_* versus *C*_e_, and their values are summarized in Table 1. Additionally, the constants of *K_f_* and n for the Freundlich isotherm model were also calculated and their values are also listed in Table 1.

As can be seen from Table 1, the Langmuir correlation coefficients (*R*^2^) for the RR120, RY167, and RB222 were 0.9813, 0.9953, and 09823, respectively, which were higher than the Freundlich correlation coefficients (*R*^2^) for the reactive dyes. Therefore, the adsorption of RR120, RY167, and RB222 onto the CL powders can be well described by the Langmuir isotherm models. Moreover, the *R*_l_ values calculated for RR120, RY167, and RB222 were found to be 0 < *R*_l_ < 1, suggesting that the adsorption of reactive dyes onto the CL powders occurs spontaneously [52]. In addition, according to the Langmuir equation, the calculated maximum adsorption capacity of RR120, RY167, and RB222 onto the CL powder were 167.0, 178.9, and 129.6 mg·g^−1^, respectively. Hence, the excellent adsorption capacity of CL powders can mainly be ascribed to the strong electrostatic interactions between the positively charged CL powder and negatively charged dye molecules under acid conditions. As shown in Figure 6f, after the adsorption process, the adsorbed CL powders were collected by the extraction filtration method under negative pressure, followed by drying in an oven at room temperature.

### 3.3. Adsorption Kinetics

**Figure 7:**
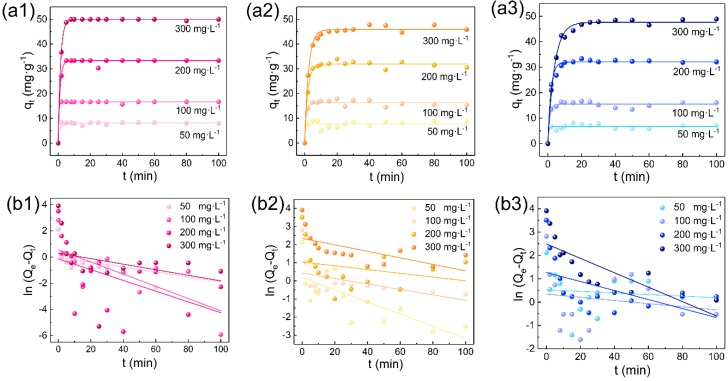
Effect of contact time on the adsorption of (**a1**) RR120, (**a2**) RY167, and (**a3**) RB222, respectively (m = 0.3 g, V = 50 mL, C_0_ = 50, 100, 200, and 300 mg·L^−1^, T = 30 °C, pH = 3.0); pseudo-first order kinetics for the adsorption of (**b1**) RR120, (**b2**) RY167, and (**b3**) RB222 onto the CL powders, respectively (m = 0.3 g, V = 50 mL, C_0_ = 50, 100, 200, and 300 mg·L^−1^, T= 30 °C, pH = 3.0).

Figure 7 shows the adsorption rate of RR120, RY167, and RB222 onto the CL powders. It is clear that all dyes exhibited rapid adsorption onto the CL powders during the first 5 min in the initial dye concentration range of 50 to 300 mg·L^−1^, which is mainly due to the strong electrostatic attraction between the adsorbate and adsorbent as well as the high dye concentration difference between the interior and exterior of the adsorbent [16]. Additionally, the active binding sites on the CL powders were quickly occupied by the dyes in a random manner, which contributed to the rapid enhancement in the adsorption process. After that, the adsorption equilibrium was obtained within approximately 10 min, which can be ascribed to the increased surface coverage of the CL powders [55]. At this stage, most of the dyes were absorbed onto the CL powders with the removal efficiency of more than 95% at the initial dye concentration range of 50 to 300 mg·L^−1^.

To further understand the adsorption mechanism of the CL powders toward the investigated dyes, three kinetic models including the pseudo-first order, pseudo-second order, and intraparticle diffusion kinetic models were considered. These kinetic models are expressed as Equations (6)–(8), respectively [57,58,59,60,61].

(6) $$\ln(Q_{e}-Q_{t})=\ln Q_{t}-k_{1}t$$

(7) $$\frac{t}{Q_{t}}=\frac{1}{k_{2}Q_{e}^{2}}+\frac{t}{Q_{e}}$$

(8) $$Q_{t}=k_{p}t^{1/2}+C$$

where *k_1_* (g·mg^−1^·min^−1^) and *k_2_* (g·mg^−1^·min^−1^) are the rate constants of the pseudo-first order and second order models; *Q_e_* (mg·g^−1^) and *Q_t_* (mg·g^−1^) are the adsorption capacities at equilibrium and at time *t* (min); h (mg·g^−1^·min^−1^) is the initial adsorption rate of the pseudo-second order kinetics; *k_p_* (g·mg^−1^·min^−1^) is the rate constant of the intraparticle diffusion model; and *C* is a constant characterizing boundary layer thickness.

**Figure 8:**
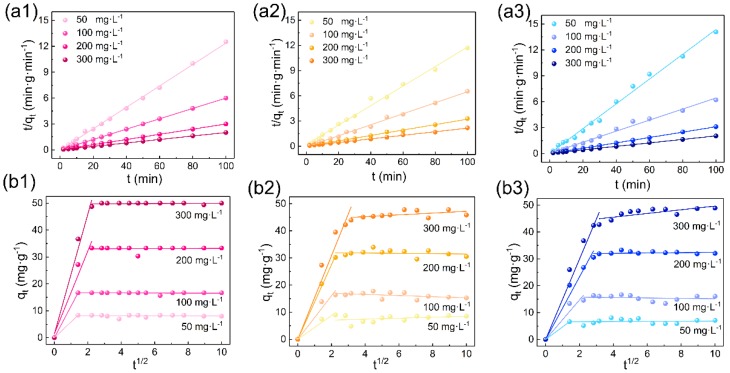
Pseudo-second order kinetics for the adsorption of (**a1**) RR120, (**a2**) RY167, and (**a3**) RB222 onto the CL powders, respectively (m = 0.3 g, V = 50 mL, C_0_ = 50, 100, 200, and 300 mg·L^−1^, T = 30 °C, pH = 3.0); intraparticle diffusion kinetics for the adsorption of (**b1**) RR120, (**b2**) RY167, and (**b3**) RB222 onto the CL powders, respectively (m = 0.3 g, V = 50 mL, C_0_ = 50, 100, 200, and 300 mg·L^−1^, T = 30 °C, pH = 3.0).

Figure 7 and Figure 8a display the plots of ln(*Q*_e_−*Q*_t_) versus t for the pseudo-first order and the plots of t/q_t_ versus t for the pseudo-second order for the absorption of RR120, RY167, and RB222 at the initial dye concentrations ranging from 50 to 300 mg·L^−1^. It is obvious that the plots were better fitted in Figure 8a1–a3 for the absorption of RR120, RY167, and RB222 by the CL powders than in Figure 7b1–b3. Moreover, from the correlation coefficient (*R*^2^) values summarized in Table 2, the pseudo-second-order model of the investigated dyes with different initial dye concentrations was higher than 0.99. Furthermore, as listed in Table 2, the theoretical adsorption capacity (*Q_e, cal_*) obtained from the pseudo-second order model was closer to the actual adsorption capacity (*Q_e, exp_*). Therefore, the pseudo-second order model is very suitable for explaining the dye removal from the wastewater by the CL powders. On the other hand, as can be seen from Figure 8b1–b3, the intraparticle diffusion was conducted by a two-step adsorption that requires separate analysis. During the first step, the adsorption of reactive dyes increased sharply, suggesting that the reactive dyes such as RR120, RY167, and RB222 diffused rapidly from the dye liquors to the external surface of the CL powders. After reaching the external surface of the CL powders, these dye molecules were attached to the accessible active sites through electrostatic attractions. After that, during the second step, the surface of the CL powders became saturated with the reactive dyes, leading to their constant diffusion.

Based on these results, it can be concluded that the CL powders showed excellent capability to remove reactive dyes from water. Additionally, the adsorption mechanism of the reactive dyes onto the CL powders is rather complex, as both the external surface adsorption and intraparticle diffusion occurred simultaneously [56,62].

### 3.4. Comparison of CL with Other Powders

**Figure 9:**
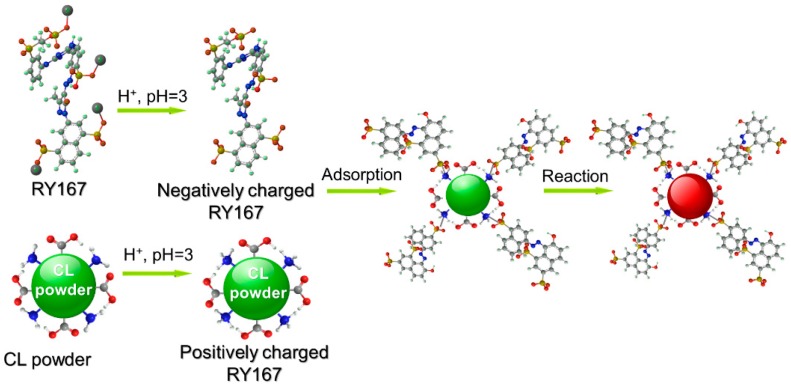
Proposed mechanism for the adsorption of reactive dyes onto the CL powders.

Table 3 summarizes the maximum adsorption capacity of dyes by other low-cost powders. It shows that the CL powders prepared in this study had a superior adsorption capacity when compared with various previously reported powders. Figure 9 shows the proposed mechanism for the adsorption of reactive dyes onto the CL powders. Due to the ionization of sulfonic acid groups in the dye molecules, the RR120, RY167, and RB222 dye molecules are generally negatively charged in the acid aqueous solution [5]. However, the CL powders are mainly composed of amino acids, and there are many functional groups such as amino groups and carboxyl groups in the CL powders. Under acidic conditions, the inhibition for the ionization of carboxyl group contributes to the enhancement of positive charge on the CL powders. Therefore, CL powders are positively charged in an acidic aqueous solution. As a result, the electrostatic attraction between the negatively charged dye molecules and the positively charged CL powders will form, leading to the adsorption of reactive dyes onto the CL powder. Simultaneously, the adsorbed reactive dye molecules can be reacted with functional groups such as amino groups on the macro chains of CL powders via a nucleophilic substitution reaction for dye fixation, thus leading to the further adsorption of reactive dyes from bulk solution onto the CL powders. Based on the above results, the CL powders can be a promising adsorbent for the effective removal of dyes from wastewater.

## 4. Conclusions

In summary, fine CL powders were successfully prepared using a homemade instrument at room temperature and atmospheric pressure. Dot-like particles with an average MFD of 1.6 μm, rod-like particles with an average MFD of 17.8 μm, and fiber-like particles with an average MFD of 40.1 μm were achieved without a separation process. The CL powders exhibited a similar chemical structure and slightly lower crystallinity when compared with CL, which was confirmed using FTIR and XRD, respectively. An improvement in thermal stability was also observed for the CL powders. This method provides an alternative avenue for the reuse of primary and secondary leather waste. Moreover, dye removal experiments indicate that the prepared CL powders could be used for the effective removal of RR120, RY127, and RB222 with the adsorption capacity of 167.0, 178.9, and 129.6 mg·g^−1^, respectively. The adsorption of reactive dyes onto the CL powders obeyed the Langmuir isotherm, and the pseudo-second-order and intraparticle diffusion models could well depict the adsorption kinetics. Therefore, the as-prepared CL powders can be used as a potential adsorbent in the treatment of dye contaminated wastewater. Future work will mainly focus on the application of the adsorbed CL powders for the pigment printing of textile materials.

## Figures and Tables

**Table 1:** Langmuir and Freundlich isotherm parameters for the adsorption of RR120, RY167, and RB222 onto the CL powders.

Dyes	Langmuir Isotherm			Freundlich Isotherm		
	*Q_m_* (mg·g^−1^)	*K_l_* (L·mg^−1^)	*R* ^2^	*K_f_* (mg·g^−1^) (L·mg^−1^)^1/n^	*1/n*	*R* ^2^
RR120	167.0	0.0229	0.9813	7.5268	0.5548	0.9231
RY167	178.9	0.0174	0.9953	6.4474	0.5946	0.9863
RB222	129.5	0.0300	0.9824	8.0707	0.4877	0.9622

**Table 2:** Kinetic parameters of the pseudo-first order and pseudo-second order kinetic models for the adsorption of RR120, RY167, and RB222 onto the CL powders.

*C_0_* (mg·L^−1^)	Dyes	*Q_e,exp_* (mg·g^−1^)	Pseudo-First Order			Pseudo-Second Order		
			*k_1_* (min^−1^)	*Q_e,cal_* (mg·g^−1^)	*R* ^2^	*k_2_* (g·mg^−1^·min^−1^)	*Q_e,cal_* (mg·g^−1^)	*R* ^2^
50	RR120	7.9861	0.0187	1.0179	0.3029	0.4502	8.0626	0.9984
	RY167	8.5526	0.0336	1.1972	0.5204	0.0706	8.6118	0.9920
	RB222	7.1078	0.0034	1.6949	0.1497	0.1909	6.9818	0.9934
100	RR120	16.667	0.0467	1.7518	0.5170	0.4081	16.667	0.9999
	RY167	15.278	0.0151	1.5394	0.1910	0.0602	15.258	0.9956
	RB222	16.071	0.0068	1.4082	0.2124	0.0831	15.654	0.9893
200	RR120	33.378	0.0411	1.1530	0.5745	0.3771	33.378	0.9999
	RY167	30.503	0.0104	2.8279	0.1709	0.0518	30.503	0.9973
	RB222	32.068	0.0193	3.5523	0.2061	0.0222	32.227	0.9996
300	RR120	50.000	0.0213	1.3612	0.2770	0.2391	49.900	0.9999
	RY167	45.856	0.0180	10.457	0.3515	0.0306	45.856	0.9984
	RB222	48.918	0.0309	12.086	0.5965	0.0113	49.617	0.9992

**Table 3:** Maximum adsorption capacity (*Q*_m_) of dyes onto the powders.

Adsorbent	Dyes	*Q*_m_ (mg·g^−1^)	Ref.
Leather powder	Reactive red 120 Reactive yellow 167 Reactive blue 222	167.0 178.9 129.5	This work
Banana peel powder	Reactive black 5	49.20	[8]
	Congo red	164.6	
Silk fibroin powder	Methylene blue	20.58	[16]
Down powder	Acid brilliant scarlet 3R	147.7	[32]
Mango seed kernel powder	Methylene blue	142.9	[63]
Neem tree leaf powder	Methylene blue	30.66	[64]
